# INCREASING ACTIVITY-STIMULATING BEHAVIOR IN DUTCH HOME CARE: FEASIBILITY OF THE SELF-PROGRAM

**DOI:** 10.1093/geroni/igad104.1118

**Published:** 2023-12-21

**Authors:** Silke Metzelthin, Lotte Hermens, Getty Huisman - De Waal, Stan Vluggen, Janneke Man - van Ginkel, Sandra Zwakhalen

**Affiliations:** Maastricht University, Care and Public Health Research Institute, Maastricht, Limburg, Netherlands; Maastricht University, Maastricht, Limburg, Netherlands; Radboud University Medical Center, Nijmegen, Gelderland, Netherlands; Zuyd Hogeschool University of Applied Sciences, Kerkrade, Limburg, Netherlands; Leiden University Medical Center, Leiden, Zuid-Holland, Netherlands; Maastricht University, Maastricht, Limburg, Netherlands

## Abstract

The concept of aging-in-place has become increasingly popular worldwide, including the Netherlands. However, as individuals age, their ability to perform activities of daily living often declines leading towards a need for homecare. Unfortunately, staff often unnecessarily take over care tasks and thereby potentially accelerate older adults’ functional decline. SELF is an interactive, tailored, holistic and theory-grounded training program consisting of 7 sessions spread over a period of 14 weeks, which aims to change staffs’ behavior towards more activity stimulating behavior. The program was evaluated in a cluster-randomized trial in Dutch nursing homes. However, its feasibility in the homecare setting was not yet known. Therefore, a feasibility study in one Dutch homecare team was conducted using a mixed-methods approach combining quantitative (i.e. checklist, logbooks, attendance forms and questionnaires) and qualitative data (i.e. focus group interviews) among program participants, team manager, coaches and trainers. Data was collected based on the three main pillars of the MRC framework: implementation, mechanisms of impact and contextual factors. In total, 24 staff members participated in the study, who delivered care to 145 clients. The SELF-program was largely implemented as intended, resulting in positive changes in staff knowledge, self-efficacy, and skills. However, contextual factors influenced the implementation of activity stimulating behaviour in practice. While reporting in electronic patient records and manager support were identified as facilitators, resistance from clients, bad expectation management by hospitals and GPs and time constraints acted as barriers. Overall, the SELF-program was feasible in Dutch homecare and only small program modifications are needed.

